# Vestibular function in degenerative parkinsonism

**DOI:** 10.3389/fneur.2026.1927218

**Published:** 2026-09-23

**Authors:** Pınar Özçelik, Koray Koçoğlu, Hatice Eraslan Boz, Müge Akkoyun, Beril Dönmez Çolakoğlu, Raif Çakmur, Gabor Michael Halmagyi, Gulden Akdal

**Affiliations:** 1 Department of Neurology, Faculty of Medicine, Dokuz Eylul University, İzmir, Türkiye; 2 Department of Neuroscience, Health Science Institute, Dokuz Eylül University, İzmir, Türkiye; 3 Department of Neurology, Royal Prince Alfred Hospital Sydney, Faculty of Medicine and Health, The University of Sydney, Sydney, NSW, Australia; 4 Central Clinical School, Faculty of Medicine and Health, University of Sydney, Sydney, NSW, Australia

**Keywords:** multiple system atrophy, Parkinson’s disease, progressive supranuclear palsy, vestibular evoked myogenic potential (VEMP), video head impulse test

## Abstract

**Introduction:**

The degenerative pathology in Parkinson’s disease (PD), multisystem atrophy (MSA) and progressive supranuclear palsy (PSP) have different spatial distribution in the brainstem. Our aim was find out in what way this pathology affects brainstem vestibular reflex pathways in each of these diseases.

**Methods:**

We studied 30 patients: 15 with PD, 9 with PSP (Richardson syndrome) and 6 with MSA (cerebellar) and compared their results with results from 37 age-matched healthy controls (HC). Motor functions of the patient groups were evaluated with the Unified Parkinson’s Disease Rating Scale (UPDRS) motor scale. We evaluated the vestibulo-ocular reflex (VOR) with the video Head Impulse Test (vHIT) and the vestibulo-collic reflex with the cervical Vestibular Evoked Mygenic Potential (cVEMP).

**Results:**

We found that PD group have reduced VOR gain in posterior and lateral SCCs and reduced cVEMP amplitude. MSA group have reduced VOR gain in posterior SCCs and reduced cVEMP amplitude while PSP group have normal VOR gain in all SCCs and normal cVEMP amplitude.

**Conclusion:**

We show that brainstem vestibular reflex pathways impaired in PD and in MSA but not in PSP by obtaining reduced VOR gain and cVEMP amplitudes. We suggest these findings are due to the presence of synuclein pathology in the lower brainstem and in vestibular nucleus in these diseases.

## 1 Introduction

The causes of neurodegenerative parkinsonism include Parkinson’s disease (PD), progressive supranuclear palsy (PSP), multisystem atrophy (MSA), Lewy body dementia and corticobasal degeneration (1). Imbalance, gait disturbance and postural instability are core axial symptoms of parkisonian syndromes and have different patterns in each (2).

The neuropathological hallmark of PD is the presence of Lewy bodies and Lewy neurites and their primary components are α-synuclein and ubiquitin (3). According to Braak’s classification, α-synuclein pathology shows a spatial distribution over time (4). MSA is also included in the synucleinopathies and α-synuclein pathology has been shown as olivopontocerebellar degeneration in MSA with predominant cerebellar ataxia (MSA-C) and striatonigral degeneration in MSA with predominant parkinsonism (MSA-P) (5).

PSP is one of the tauopathies, a form containing four repeats (4-repeat tau-4R tau), characterized by the accumulation, neuronal loss and gliosis (6). PSP subtypes show different pathological distribution in the brainstem (7).

Frequent falls and imbalance are important factors that effects the function and health quality of patients with parkinsonism. Not only motor system but autonomic dysfunction, sleep disturbance and cognitive impairment contribute balance and falls in PD patients (8). The contribution of vestibular dysfunction to imbalance in parkinsonism, especially in PD, has become recognized and vestibular system evaluation clinical practice has been recommended (9). Galvanic vestibular stimulation has been shown to have positive effect of static postural balance of PD patients (10, 11). Here we explore if brainstem vestibulo-ocular or vestibulo-collic reflexes are impaired in PD, MSA and PSP.

## 2 Methods

We studied 15 PD, 9 PSP Richardson’s syndrome and 6 MSA-C diagnosed patients and 37 healthy controls (HCs). PD patients were diagnosed as ‘clinically established Parkinson’s disease’ according to Movement Disorders Society 2015 diagnostic criteria (12). MSA patients were diagnosed as ‘probable MSA’ and PSP patients were diagnosed as ‘probable PSP’ according to MDS diagnostic criteria (13, 14).

The exclusion criteria were diagnosis of vascular, drug-induced, or metabolic parkinsonism; inability to comply with the tests for any reason; and a known history of another neurological or psychiatric disorder. None of the patients had known vestibular disorder and history of hearing loss. HCs were volunteers who had no vestibular or neurologic disorder and history of hearing loss.

Motor domain of Unified Parkinson’s Disease Rating Scale (UPDRS) was used to determine clinical status of the patients (15). All patients with PD included in the study were receiving L-dopa treatment. Patients with MSA and PSP were receiving levodopa treatment at doses that were being adjusted according to their clinical status. PSP group were receiving either L-dopa or amantadine while MSA were receiving L-dopa treatment. All patients with Parkinson’s disease were evaluated during the medication “on” state, corresponding to the period of maximal clinical benefit from their usual antiparkinsonian medication.

We used the cervical vestibular evoked myogenic potential (c-VEMP) test to investigate the vestibulo-collic reflex and video head impulse test (vHIT) to investigate the vestibulo-ocular reflex (VOR). Participants in the patient group who were unable to achieve the target SCM contraction range required for VEMP testing, and those who were unable to maintain the appropriate position for vHIT testing due to rigidity were also excluded from the study. The study protocol was approved by the clinical research ethics committee of Dokuz Eylul University protocol number was 6,338-GOA and approval number was 2021/14–28. All of the participants in the study signed informed written consent.

### 2.1 VEMP

We tested c-VEMP with Eclipse EP25 system® (Interacoustics AS, Middelfart, Denmark). The external auditory canals were examined for any conditions that could interfere with the delivery of the acoustic stimulus. Audiometry was not performed.

Monaural air-conducted (AC) tone burst stimuli via RadioEar IP30 insert headphones and bone-conducted (BC) tap stimuli via Radioear type B71 bone vibrator to mastoid were used to evoke potentials. The bone-conducted stimulator was calibrated according to the manufacturer’s factory calibration specifications. Contralateral head turning while participant in sitting position was used to contract SCM muscle in cVEMP testing. The target electromyographic (EMG) level during sternocleidomastoid (SCM) muscle contraction was set at 75–150 μV. Evoked potentials were averaged over 200 repetitions. Following the recording of the amplitude values in μV, EMG rectification was performed to account for the influence of background muscle activity on the recorded responses. The rectified averaged prestimulus EMG less than 2 microvolts. In our study, the rectification procedure was performed automatically by the device using the method of dividing the averaged amplitude values recorded in μV by the square root of the pre-stimulus EMG activity (16).

cVEMPs are inhibitory responses generated by SCM muscle contraction, and their amplitudes are influenced by the level of muscle activation (17). This is particularly relevant in patients with parkinsonism, in whom neck rigidity may affect SCM activation during testing. Although all participants were tested within the predefined EMG activity range, differences in SCM activation were observed, particularly in the parkinsonism group. Therefore, both raw and EMG-rectified amplitudes were presented, while the EMG-rectified results were used for interpretation.

cVEMP response was considered present when a reproducible biphasic p13–n23 waveform was identified in at least two consecutive recordings with adequate SCM muscle activation. A response was considered absent if a reproducible response could not be obtained, defined as the absence of time-independent potentials >5 μV, or if no response was elicited in two repeated recordings.

Five hundred Hz stimuli frequency with 97–100 db-NHL intensity for cVEMP and plateau time was 2 ms for cVEMP. EMG band-pass filtered between 10 and 1,000 Hz.

### 2.2 vHIT

We tested vHIT in the 6 individual semicircular canals (SCCs) to evaluate VOR using GN Otometrics apparatus (ICS Impulse, GN Otometrics, Taastrup Denmark). The participant is asked to look at the target 1 m away and at least 20 impulses with unpredictable time and direction are delivered in each SCC direction. Head impulses are performed by turning the participant’s head about 20 ° with peak velocity of 100–300 °/s. The minimum peak velocity of the head impulses was >120°/s (18, 19).

### 2.3 Analysis of data

vHIT: Individual VOR gains were calculated as the area ratio beneath each eye and head velocity curve. Artifacts were detected and removed semi-automatically from each impulse eye velocity profile. Following the procedure, the recordings were repeated when eyelid artifacts or findings suggestive of google slippage were identified, and the test was repeated until these artifacts were eliminated (20). Head impulses yielding extremely low or high VOR gain values that could substantially affect the distribution of the averaged responses were manually excluded from the analysis.

Vestibulo-ocular reflex gain values obtained from the right and left SCCs were averaged for lateral, posterior and anterior planes. All head impulse test results were assessed for the presence of corrective saccades. The presence of corrective saccades was compared between groups for each plane. Low VOR gains were considered as **≤** 0.80 for horizontal SCCs, **≤** 0.70 for vertical SCCs (21).

VEMP: The initial positivity and following first negativity of the responses were marked as p and n, respectively, for cVEMP. Latency was measured for each p and n point. Raw amplitudes were determined as peak-to-peak amplitude (22). Amplitudes and latencies were measured for the left and right ear and mean values of the responses were obtained to compare groups.

### 2.4 Statistics

Statistical analyses were performed using IBM SPSS Statistics for Windows, Version 29.0 (IBM Corp., Armonk, NY, USA). To address the non-independence of bilateral measurements, data from the right and left ears or SCCs were averaged for each participant, yielding a single value per subject; thus, the individual participant served as the primary statistical unit.

The normality of continuous variables was evaluated using the Kolmogorov–Smirnov and Shapiro–Wilk tests. As the data were not normally distributed, continuous variables were presented as Median (Interquartile Range, IQR). Comparisons of continuous variables across the four groups (HCs, PD, PSP, and MSA) were performed using the Kruskal–Wallis H test. For parameters showing significant differences in the Kruskal–Wallis test, pairwise post-hoc subgroup comparisons were conducted using Dunn’s test with Bonferroni correction to adjust for Type I error inflation in multiple testing. Effect sizes for Kruskal–Wallis tests were calculated using H-based eta-squared ( $\eta_{H}^{2}=\frac{H-k+1}{N-k}$ ). Post-hoc pairwise comparisons were performed for all outcomes using Dunn’s test with Bonferroni correction, and only statistically significant pairwise comparisons are presented in the results section.

Categorical variables were compared across groups using the Chi-square test, or Fisher’s exact test when expected cell counts were less than 5.

Given the small sample sizes of rare parkinsonian syndromes (MSA-C and PSP), this study was designed as exploratory in nature. The sample size was calculated using OpenEpi based on the difference in mean VEMP amplitudes between PD and HCs reported by de Natale et al. (23). A sample size of 30 patients and 30 HCs was required to achieve 80% power at a 95% confidence level. Statistical significance was defined as a two-tailed *p*-value < 0.05.

## 3 Results

There were no differences between groups in terms of age, gender, disease duration and UPDRS motor scale (Table 1).

**Table 1:** Demografic and clinical features of the groups.

	HCs median(IQR)	PD median(IQR)	PSP median(IQR)	MSA median(IQR)	Kruskal–Wallis *H*	*p*
Age	63.0(11.0)	68.0(12.0)	65.0(10.5)	62.0(11.5)	3.133	0.372**a**
Gender	22F/15M	5F/10M	4F/5M	3F/3M		0.390**b**
Disease duration (Month)	--	36.0(70.0)	24.0(42.0)	18.0(12.0)	4.646	0.098**a**
UPDRS motor total	--	32.0(32.0)	38.0(31.0)	48.5(23.5)	4.925	0.085**a**

HCs: Healthy Controls, PD: Parkinson Disease, PSP: Progressive supranuclear palsy, MSA: Multisystem atrophy, **ᵃ**Kruskall Wallis Test, **ᵇ**Fisher exact test.

### 3.1 vHIT and VEMP results

PD group have reduced VOR gain in posterior and lateral SCCs and reduced cVEMP amplitude than HCs. MSA group have reduced VOR gain in posterior SCCs and reduced cVEMP amplitude than HCs. The PSP group did not differ significantly from the HCs in terms of vHIT and cVEMP findings. These results are shown in Tables 2 3.

**Table 2:** vHIT results of the groups.

**VOR gain of SCCs**	**HCs**	**PD**	**PSP**	**MSA**	**Kruskal–Wallis H**	*p*-value	Adjusted *p*-value*
	*Median (IQR)*	*Median (IQR)*	*Median (IQR)*	*Median (IQR)*			
VOR gain – lateral SCCs	0.99 (0.19)	0.84 (0.22)	0.99 (0.46)	0.85 (0.43)	9.937	**0.019ᵃ**	HCs-PD:0.002
VOR gain – anterior SCCs	0.88 (0.13)	0.81 (0.26)	0.86 (0.35)	0.81 (0.19)	3.920	0.270ᵃ	–
VOR gain – posterior SCCs	0.79 (0.15)	0.70 (0.16)	0.75 (0.25)	0.69 (0.18)	9.190	**0.027ᵃ**	HCs-PD:0.009 HCs-MSA: 0.047
	*n (percentage)*	*n (percentage)*	*n (percentage)*	*n (percentage)*			
Overt saccade lateral SCCs							
Positive	9 (24.3%)	7 (46.7%)	3 (33.3%)	2 (33.7%)		0.467ᵇ	
Negative	28 (75.7%)	8 (53.3%)	6 (66.7%)	4 (66.7%)			
Overt saccade anterior SCCs							
Positive	0	0	0	0		--	
Negative	37 (100%)	15 (%100)	9 (%100)	6 (%100)			
Overt saccade posterior SCCs							
Positive	0	2 (13.3%)	0	0		0.136ᵇ	
Negative	37 (100%)	13 (86.7%)	9 (100%)	6 (100%)			

HCs: Healthy Controls, PD: Parkinson Disease, PSP: Progressive supranuclear palsy, MSA: Multisystem atrophy, SCCs: Semicircular canals, VOR: Vestibulo-ocular reflex, ᵃ Kruskall Wallis Test, ᵇ Fisher exact test.

**Table 3:** VEMP results of the groups.

	HCs	PD	PSP	MSA	Kruskal–Wallis *H*	*p-*value	Adjusted *p*-value *
	*Median (IQR)*	*Median (IQR)*	*Median (IQR)*	*Median (IQR)*			
c-VEMP/AC amplitude raw	100.61(88.90)	51.16 (26.99)	50.93 (37.37)	49.81 (−)	12.80	**0.005a**	HCs-PD = 0.025 HCs-PSP = 0.026 HCs-MSA = 0.015
c-VEMP/AC amplitude corrected	0.88 (0.64)	0.42 (0.22)	0.36 (0.42)	0.42 (−)	3.920	**0.035a**	HCs-PD = 0.049
c-VEMP/BC amplitude raw	75.92 (82.47)	44.39(22.57)	62.84(89.41)	36.99(44.71)	9.190	**0.005a**	HCs-PD = 0.004 HCs-MSA = 0.010
c-VEMP/BC amplitude corrected	0.65 (0.48)	0.44 (0.15)	0.49 (0.57)	0.30 (0.46)	9.198	**0.027a**	HCs-PD = 0.011 HCs-MSA = 0.038
c-VEMP AC latency	P:15.50 (1.18)	P:14.50 (2.92)	P:15.00 (2.46)	P:14.34 (−)	1.093	0.779**a**	
	N:24.34 (2.34)	N:22.33 (9.04)	N:23.08 (1.83)	N:21.66 (−)	5.615	0.132**a**	
c-VEMP BC latency	P:16.75 (2.88)	P:17.17 (3.66)	P:16.33 (3.16)	P:16.83 (3.42)	1.042	0.791**a**	
	N:25.92 (6.00)	N:26.00 (3.16)	N:25.00 (3.97)	N:24.00 (7.75)	2.186	0.535**a**	
	*n(percentage)*	*n(percentage)*	*n(percentage)*	*n(percentage)*			
c-VEMP/AC							
Response present	32 (86.5%)	8 (53.3%)	7 (22.2%)	3 (50%)		**0.031b**	HCs-PD: 0.016
Response absent	5 (13.3%)	7 (46.7%)	2 (77.8%)	3 (50%)			
c-VEMP/BC							
Response present	34 (91.9%)	11 (73.3%)	9 (100%)	5 (83.3%)		0.169**b**	
Response absent	3 (8.1%)	4 (26.7%)	0	1 (16.7%)			

HCs: Healthy Controls, PD: Parkinson Disease, PSP: Progressive supranuclear palsy, MSA: Multisystem atrophy. **a**Kruskall Wallis Test, 𝑿isher exact test, AC: Air Conducted, BC: Bone Conducted.

Reduced VOR gains were identified in the lateral SCCs of 4 patients with PD and 1 patient with MSA, in the anterior SCCs of 2 patients with PD and 1 patient with MSA, and in the posterior SCCs of 6 patients with PD and 3 patients with MSA. The presence of overt saccades did not differ significantly among the groups (Table 2).

When the groups were compared in terms of response presence following air-conducted (AC) stimulation, absent responses were more frequent in the PD group than in the HCs. The results of the BC stimulation did not differ between groups (Table 3).

## 4 Discussion

We found that c-VEMP amplitudes of the PD and MSA groups were lower than those of HCs. We suggest that these results are related to the spread of synucleinopathy, the pathological process of PD and MSA. Braak’s theory of spread, which suggests that the pathology in PD begins in the lower brainstem, in the medulla, spreads to the pons and then to the cortical areas, explains the effect on the vestibulo-collic reflex and the decrease in amplitude or lack of response in c-VEMP (4). Additionally, degeneration of the venterolateral medulla occurs in MSA, and also in olivopontocerebellar and nigrostriatal degeneration (23). However, the pathology of PSP begins in the pedunculo-pontine nucleus in the mesencephalon; involvement of the lower brainstem and medulla oblongata occurs in later stages (24). However, given the relatively small sample size of the present study, these interpretations should be considered hypothesis-generating rather than definitive evidence of a specific pathophysiological mechanism.

Among patients with degenerative parkinsonism, PD is the most extensively studied group in terms of VEMP responses. In a meta-analysis published by Cui et al. in 2022, the findings of 15 studies comparing patients with PD and healthy controls were pooled. The analysis demonstrated that absent cVEMP responses were more frequent in patients with PD than in HCs (25).

We found that more participants in all groups respond to BC stimulation than AC stimulation in cVEMP. AC stimulation is generally the preferred method in c-VEMP because the saccule seems to be more sensitive to AC than BC stimulation (26). However the response to AC stimulation decreases with increasing age and BC stimulation can elicits a c-VEMP response, is absent with AC stimulation in the age range of our study group. c-VEMP responses have been obtained from more individuals with BC stimulation in both PD and HCs (27), as we found in our study.

We found decreased VOR gain from the lateral SCCs in PD and from the posterior SCCs in PD and MSA. vHIT results in diseases affecting the central vestibular system have shown that acute or chronic vestibular nuclear and cerebellar impairment can reduce VOR gain (28). An acute unilateral vestibular nuclear infarction can cause decrease of lateral and posterior SCC vHIT gain (29, 30). This could be due to the relative preservation of the anterior canals, as the lateral and posterior SCC carry their signals primarily to the medial vestibular nucleus, while the anterior SCC carries them to the superior and medial vestibular nucleus (31). However, this anterior SCC sparing pattern has not been demonstrated in chronic progressive cerebellar ataxias, and it has been suggested that widespread damage may be observed in all SCCs in the course of degenerative disease (32). vHIT evaluation in patients with spinocerebellar ataxia type 6 showed that anterior SCC gain increased at the beginning of the disease perhaps due to the loss of anterior SCC inhibition by the flocculus but decreased later in the disease, and posterior SCC gain remained low from the beginning of the disease (33). On the other hand asymmetry in vertical SCC gain between anterior and posterior SCC VOR gain was larger in MSA than PD (34).

Hawkins et al. evaluated vHIT in PD and HCs and found no difference in VOR gain between them (27). They suggested that the PD patients included in their study were in mild or moderate stages, and VOR impairment was not observed in these stages. However, the two advanced-stage PD patients in their study had low VOR gain and corrective saccades, suggesting that VOR impairment occurs in the advanced stage. We found that 4 PD patients (Hoehn Yahr stage 1) had low horizontal SCCs gain and 2 of them with corrective saccades. Therefore, advanced disease stage did not explain VOR impairment in our study. Lv et al. evaluated only lateral SCC gain and surprisingly found that PD that higher VOR gain than HCs and suggested that this result could be due to a compensation mechanism for early-stage PD (35).

In addition to conflicting vHIT results in PD, there is conflicting data about VOR with caloric testing and rotary chair testing. Cipparone et al. observed vestibular hyperreflexia in 48.6% of caloric test results in the PD group, while Reichert et al. reported that one-third of participants with PD had no caloric response and a reduced response in another third (36, 37). In a more recent study, Venhoven et al. compared six MSA and three PSP patients with 30 PD patients using caloric testing and rotary chair testing, and found no significant difference in VOR gain between the groups (38). These studies are limited by the well known variability of the absolute magnitude of caloric and rotational response magnitudes in normal subjects.

Lewy bodies are found in the vestibular nucleus and in the ocular motor nuclei in PD, and in the vestibular nucleus and cerebellar Purkinje cells in MSA, especially in MSA-C which has olivopontocerebellar degeneration (39). This spread of degenerative pathology may explain our vHIT results as we found VOR impairment in PD and MSA group. As with the cVEMP responses, our interpretations of the vHIT findings should also be considered hypothesis-generating rather than definitive evidence of a specific pathophysiological mechanism. On the other hand, we did not find VOR impairment in the PSP group, as expected, due to gaze palsy is supranuclear in PSP. VOR is the last ocular motor deficit affected by the disease in PSP and is preserved until the final stages (40). The horizontal vHIT was found to be normal in 16 PSP patients (41).

### 4.1 Limitation of the study

We initially included ocular VEMP testing in our study to further assess upper brainstem reflexes. However, this test was excluded because some patients with PSP have significant upward gaze paralysis, which may have affected their responses. Another limitation of our study is the relatively small sample size; however, it was consistent with the sample size determined by the power analysis.

## Data Availability

The raw data supporting the conclusions of this article will be made available by the authors, without undue reservation.
